# The Influence of Staff-Resident Interactions on Resistiveness to Care Behavior in Assisted Living

**DOI:** 10.1093/geroni/igab046.3107

**Published:** 2021-12-17

**Authors:** Rachel McPherson, Barbara Resnick, Elizabeth Galik

**Affiliations:** 1 University of Maryland Baltimore and Baltimore County, Catonsvile, Maryland, United States; 2 University of Maryland, Baltimore, Maryland, United States; 3 University of Maryland School of Nursing, Baltimore, Maryland, United States

## Abstract

Resistiveness to care (RTC) is a behavioral and psychological symptom of dementia that is common among dementia residents in assisted living facilities. RTC encompasses verbal and nonverbal behaviors that oppose care, such as crying, grabbing, hitting, or yelling, among many other resistive behaviors. The quality of care interactions which can be positive, neutral or negative, have been associated with increased RTC. The purpose of this study was to test the association between quality of care and RTC. This was a secondary data analysis using baseline data from the Function-Focused Care for Assisted Living Using the Evidence Integration Triangle (FFC-AL-EIT) implementation study. Controlling for cognition, age, gender, medication use, and comorbidities, it was hypothesized that quality of care interactions would be associated with resistiveness to care. A linear regression analysis was conducted to test the hypothesis. The sample included 794 participants the majority of whom were white women with a mean age of 89.48 (SD=7.61). The mean RTC was .09 (SD=.41, range 0-13) and the mean quality of care interactions were 5.96 (SD=1.44, range 0-7). Based on the regression analysis there was no significant association between quality of care and RTC. These findings may be due to the high quality of care provided and limited RTC in this sample. Ongoing research is needed, however, to continue to explore these relationships and assure that all RTC is being reported among staff and that there is no evidence of negative quality of care interactions in these settings.

